# Molecular mechanisms and therapies for tumor inhibition through the arginine metabolism pathway

**DOI:** 10.3389/fonc.2026.1774392

**Published:** 2026-02-11

**Authors:** Yuhang Xu, Mingxin Yu, Yi Zhang, Yiqing Jiang, Haiyan Zhu, Shujuan He, Guohua Yu, Niannian Li, Shuzhen Liu, Bin Liu

**Affiliations:** Weifang People’s Hospital, Shandong Second Medical University, Weifang, Shandong, China

**Keywords:** arginine deprivation, arginine metabolism, inhibitor, tRNA modification, tumor treatment

## Abstract

Tumors are one of the major diseases leading to human death. Arginine metabolism plays an important role in tumor occurrence and metastasis. Based on the levels of arginine in tumor cells, methods such as recombinant arginine deiminase are used to reduce arginine in order to inhibit tumor growth. However, arginine deprivation therapy has limited efficacy in tumor cells due to increased arginine synthesis, resistance to chemotherapeutic agents, metabolic reprogramming, and the suppression of immune cells in the tumor microenvironment. Meanwhile, with the revelation of many new molecular mechanisms by which arginine controls tumor cell growth, numerous newly designed molecules targeting arginine metabolic pathways for cancer treatment have emerged. In this review, we integrate and analyze the responses of tumor cells and immune cells such as T cells to arginine and strategies for cancer therapy. At the molecular level, we review and discuss the mechanisms of specifically blocking arginine-regulated metabolic reprogramming in cancer cells, the effector factors from pathogenic microorganisms and metabolites from plants in inhibiting cancer cells via arginine metabolism, and arginine tRNA metabolic pathway. Finally, we discuss the mechanisms and case studies of using antineoplastic agents that target arginine metabolic pathways in combination. This review collects and integrates the mechanisms and experiences of treating various cancers through arginine and its metabolic derivatives, providing direct therapy guidance for cancer patients with disordered arginine metabolism in the tumor and immune cells.

## Graphical Abstract

**Figure:**
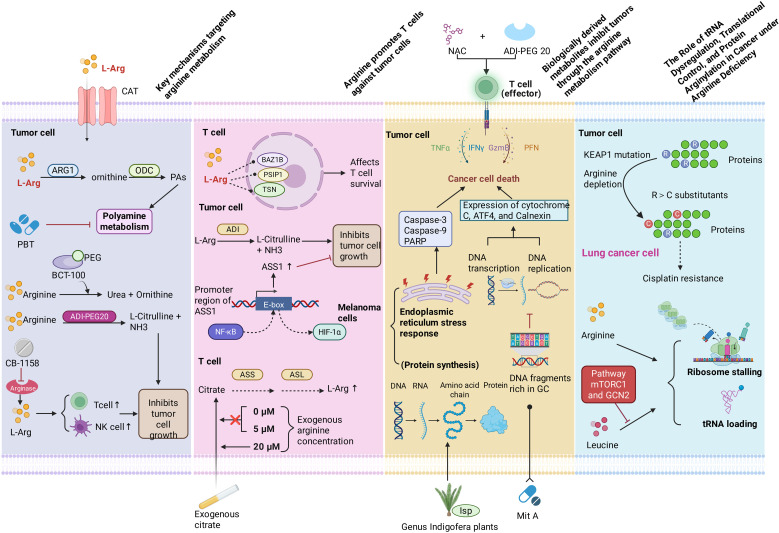
L-Arg, L-Arginine; CAT, Cationic amino acid transporter; ARG1, Arginase 1; ODC, Ornithine decarboxylase; PAs, Polyamines; ADI, Arginine Deiminase; ASS1, Argininosuccinate synthase 1; ASL, Argininosuccinate lyase; GCN2, General control non-derepressible 2; mTORC1, mammalian Target Of Rapamycin Complex 1.

## 1 Introduction

Cancer cells undergo profound metabolic reprogramming to support their uncontrolled proliferation and survival. Many studies have shown that arginine promotes tumor growth. In liver cancer cells, even when the expression of arginine synthesis genes is reduced, the cells can increase the amount of arginine to maintain tumor cell growth (1). Arginine deprivation directly leads to the death of cancer cells (2), so directly reducing the amount of arginine in tumor cells has become an important tumor treatment method, which is also known as arginine deprivation therapy (3).

The arginine metabolism axis intersects with multiple hallmarks of cancer. Intracellular arginine serves as a precursor for polyamine synthesis, supporting cancer cell growth (4, 5), and for nitric oxide (NO) production (6), influencing angiogenesis and signaling. Its depletion triggers integrated stress responses, disrupts mitochondrial function, and can induce cell cycle arrest and apoptosis specifically in ASS1-deficient tumor cells. However, resistance mechanisms, including adaptive re-expression of ASS1 (7), and the complex role of arginine in modulating the tumor immune microenvironment where it can either support T-cell function or be catabolized by myeloid-derived suppressor cells to promote immunosuppression (8), present both opportunities and challenges.

This review aims to synthesize current knowledge on the molecular mechanisms by which targeting arginine metabolism inhibits tumor progression. We provide a critical review and discussion of therapeutic strategies, from direct arginine depletion using pegylated arginine deiminase (ADI-PEG20) and recombinant human arginase to pharmacological inhibition of enzymes like arginase and nitric oxide synthase. Furthermore, we explore emerging combinatorial approaches that leverage arginine deprivation therapy to potentiate chemotherapy, radiotherapy, and immunotherapy, and ultimately discuss future directions for translating these metabolic insights into more effective and personalized anticancer regimens.

## 2 Functions of L-arginine in the human body

During infancy, growth periods, pregnancy, and illness, the body’s synthesis of arginine is insufficient to meet the cells’ demand for arginine, so dietary supplementation is needed; therefore, arginine is considered a semi-essential amino acid (9). Arginine can not only be interconverted with proline and glutamate, but also serves as a precursor for the synthesis of proteins, nitric oxide, creatine, polyamines, agmatine, and urea (10). The concentration of arginine is increased in various tumors and is a key amino acid for regulating and adapting the immune system. Its metabolic derivatives, such as NO and polyamines, participate in the occurrence and development of tumors (11). In the human body, arginine is primarily synthesized through the urea cycle (in the liver) and the cytoplasmic pathway in tissues such as the kidneys and intestines, with the kidneys being the main organ responsible for net arginine synthesis (12). Arginine metabolism occurs via multiple pathways. Arginine produces NO through the NOS pathway, regulating cardiovascular, neural, and immune functions (13); arginase catalyzes the conversion of arginine to ornithine, which is then used to synthesize polyamines and proline. Polyamines contribute to cell proliferation and repair, while proline is involved in collagen synthesis and tissue repair. Arginase I mainly detoxifies ammonia in the liver (14), whereas arginase II regulates polyamine/proline synthesis and immune balance in extrahepatic tissues (15); arginine-depleting enzymes can catalyze L-arginine to generate agmatine, which regulates neural and vascular relaxation (16); L-arginine:glycine amidinotransferase (GATM) catalyzes the transfer of a guanido group from arginine to glycine, forming guanidinoacetic acid, the immediate precursor of creatine (17); L-Arginine deiminase (ADI) catalyzes the hydrolysis of arginine to citrulline and ammonia. The main way the gut microbiota metabolizes arginine is through ADI (18).

### 2.1 Arginine biosynthesis and metabolism

**Figure 1:**
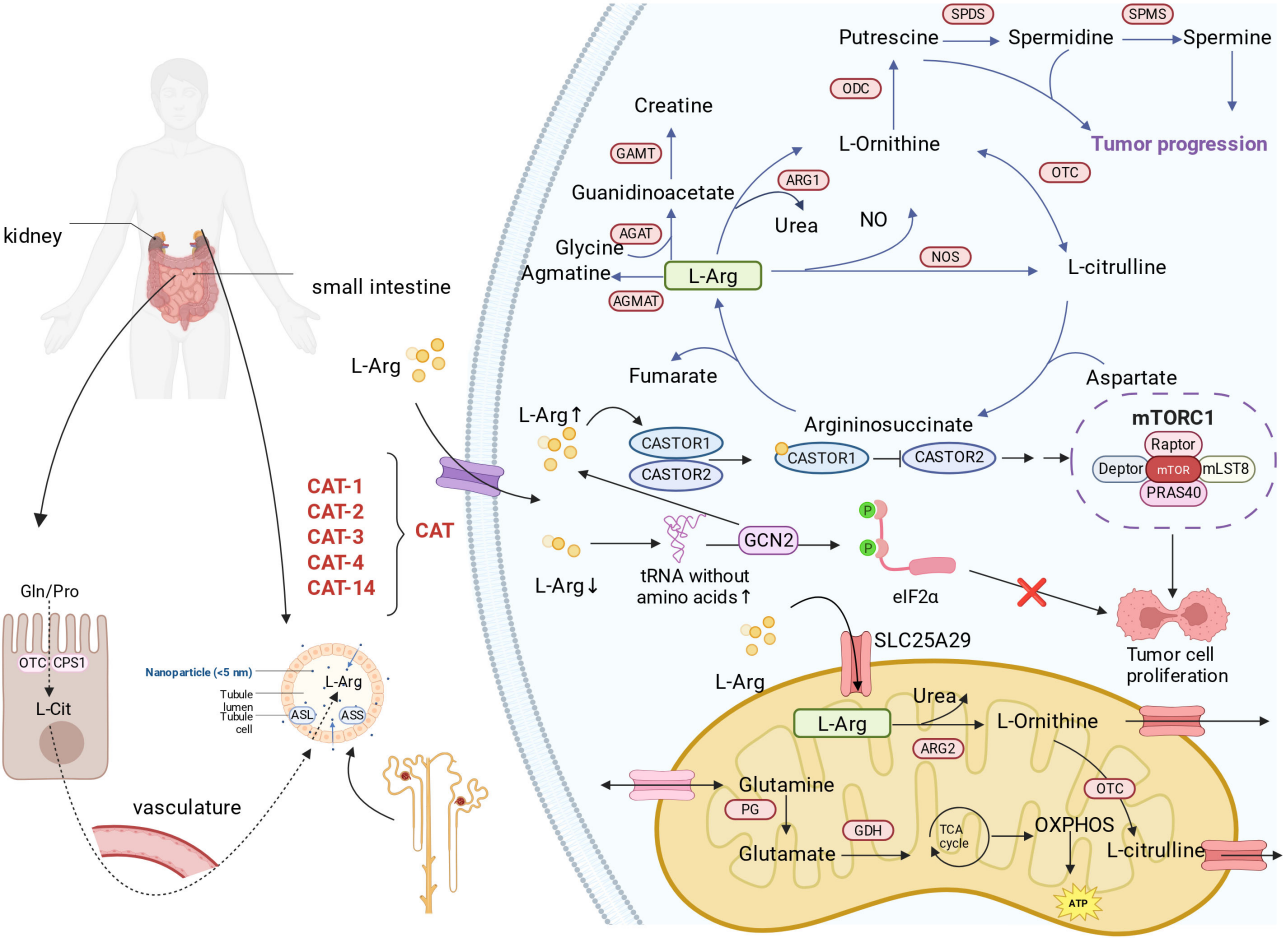
Arginine metabolism in tumor cells. It is synthesized mainly via the urea cycle and absorbed through specific transporters like SLC7A1 and SLC25A29. In tumors, arginine metabolism is reprogrammed to promote growth, immune evasion, and angiogenesis. Arginine is broken down into NO, polyamines, and creatine, influencing proliferation and metastasis of cancer cells. Arginine also acts as a signaling molecule, activating mTORC1 and GCN2 pathways to regulate metabolism and survival. L-Arg, L-Arginine; CAT, Cationic amino acid transporter; SLC25A29, Solute carrier family 25 member 29; ADC, Arginine decarboxylase; AGAT, Arginine-glycine amidinotransferase; GAMT, Guanidinoacetate methyltransferase;ARG, Arginase; ODC, Ornithine decarboxylase; OTC, ornithine transcarbamylase; NOS, Nitric oxide synthase; SPMS, Specialized pro-resolving mediators; PG, Protein Glutaminase; GDH, Glutamate dehydrogenase; GCN2, General control non-derepressible 2; mTORC1, mammalian Target Of Rapamycin Complex 1.

Carbamoyl phosphate synthetase 1 (CPS1) and ornithine transcarbamylase (OTC) are expressed in adult intestinal epithelial cells, where they convert dietary glutamine and proline into citrulline. Citrulline is absorbed by the renal proximal tubules and converted into arginine under the action of argininosuccinate synthase (ASS) and argininosuccinate lyase (ASL) (19) (**Figure 1**). Although the kidneys are the main organs where citrulline is converted to arginine in the body, up to 40% of citrulline clearance occurs in tissues outside the kidneys (20). Arginine in cells is mainly synthesized through the urea cycle (21). Carbamoyl phosphate synthetase I (CPS-I) catalyzes the synthesis of carbamoyl phosphate from ammonia and carbon dioxide with the energy provided by ATP. Ornithine transcarbamylase (OCT) transfers the carbamoyl group from carbamoyl phosphate to ornithine, producing citrulline. Argininosuccinate synthase (ASS) catalyzes the condensation of citrulline and aspartate, consuming ATP to produce argininosuccinate. Argininosuccinate is then cleaved by argininosuccinate lyase (ASL) into arginine and fumarate (**Figure 1**). At the same time, the rate of *de novo* arginine synthesis depends on the exchange rate of arginase between different cellular compartments (22).

Although human cells can synthesize arginine, the rate of synthesis is insufficient to meet the body’s needs. Therefore, humans still need to obtain arginine from their diet. Cells rely on transporters on the cell membrane to take up arginine from the extracellular environment (23). The known arginine transporters are mainly cationic amino acid transporters (24). The CAT family of transporters mainly includes CAT-1, CAT-2, CAT-3, and CAT-14, which are encoded by genes of the solute carrier family 7 (SLC7). CAT-1 (also known as SLC7A1) has a high affinity for arginine and is one of the main pathways for arginine to enter cells (**Figure 1**). CAT-2 (also known as SLC7A2) is an inducible arginine transporter and has two isoforms, CAT-2A and CAT-2B. Among them, CAT-2A has a low affinity for arginine, whereas CAT-2B is expressed in macrophages and has a very high affinity for arginine (25). CAT-3 is also involved in the transport of arginine, with an arginine affinity between the affinities of CAT-1 and CAT-2.

SLC25A29 is located on the mitochondrial membrane and belongs to the Solute Carrier Family 25 (SLC25). It can transport basic amino acids from the cytoplasm into the mitochondria. SLC25A29 transports arginine, lysine, homoarginine, methylarginine and, to a much lesser extent, ornithine and histidine (26) (**Figure 1**). *In vitro* experiments and recombinant protein studies show that SLC25A29 can perform significant uniport transport and can also participate in a counter-exchange mechanism, transferring cytosolic basic amino acids into the mitochondrial matrix to meet the needs of mitochondrial protein synthesis and arginine catabolism metabolism (26). The function of SLC25A29 can be inhibited by mitochondrial carrier inhibitors such as mercurial compounds, and its structure and function share similar characteristics with other classic members of the mitochondrial carrier family.

SLC25A29 is upregulated or abnormally expressed in various tumor types. Upregulation of SLC25A29 can increase the uptake of arginine into mitochondria, elevate the levels of nitric oxide (NO) produced within mitochondria, thereby affecting mitochondrial respiration and metabolic reprogramming, and supporting the energy metabolism and proliferation demands of cancer cells (27)(**Figure 1**). After knocking out or knocking down SLC25A29, supplementing with exogenous arginine still cannot restore the related metabolic pathways, indicating its essential role in mitochondrial arginine transport (27). In prostate cancer (especially castration-resistant prostate cancer), elevated expression of SLC25A29 is closely associated with tumor metastasis and poor prognosis (28). SLC25A29 participates in the metabolic remodeling of pancreatic cancer cells by promoting the synthesis of NO so that the cells get adapted to the harsh environment and survive (29). Other studies, such as those on lung adenocarcinoma and gastric cancer, also show that the expression of SLC25A29 is associated with tumor staging and invasion, and it involves in the regulation of endothelial cell phenotypes in the tumor microenvironment (30).

### 2.2 Arginine catabolism

There are four known arginine catabolic pathways, and arginine catabolism produces various metabolic products. Arginine catabolism includes 1) the conversion of arginine into nitric oxide (NO) and L-citrulline catalyzed by nitric oxide synthase (NOS) (31);2) Under the catalysis of arginase 1 (ARG1), arginine is converted into polyamines (32), which mainly include putrescine, spermidine, and spermine. Among them, ARG1 catalyzes arginine, converting it first into ornithine, which then further generates polyamines through the action of ornithine decarboxylase (ODC), promoting the polarization of macrophage cells toward a pro-tumor phenotype (M2 type), thereby indirectly inhibiting anti-tumor immunity (33);3) AGMAT catalyzes the conversion of arginine to agmatine, which is then further converted into polyamines; 4) Arginine and glycine, under the action of arginine glycine aminotransferase (AGAT), produce ornithine and guanidinoacetate. Ornithine and guanidinoacetate are further converted into creatine and S-adenosylhomocysteine under the action of guanidinoacetate N-methyltransferase (GAMT) (34) (**Figure 1**).

The catabolic products of arginine promote tumor growth. The increased nitrogen demand of tumor cells promotes the urea cycle (UC) reprogramming. This facilitates the efficient use of ammonia in tumor cells to synthesize UC intermediates and interact with other metabolic pathways, particularly nucleic acid and polyamine synthesis. Finally, this promotes tumor growth and progression (35). Tumor cells evade immune surveillance through ammonia metabolism (36). Polyamines promote protein and DNA synthesis. At the same time, they can stabilize tumor-associated transcription factors such as c-Myc (37). Polyamines can also inhibit apoptosis, enhancing tumor cell survival. Nitric oxide mediates angiogenesis, increases tumor blood supply, and also promotes immune evasion by inhibiting immune cell activity, while inducing accumulation of DNA damage and driving genetic mutations (38). In addition, arginine metabolism maintains the redox balance of tumor cells, such as the synthesis of glutathione, to resist oxidative stress (39).

Arginase has two isoenzymes: type I (AI) is present in the liver and is the main form of arginase, while type II (AII) is found in extrahepatic tissues and is present in smaller amounts. The arginine absorbed from the diet generates NO under the action of nitric oxide synthase (NOS), which can act as a nitrogen oxide compound and has effects such as promoting angiogenesis, tumor metastasis, anti-apoptosis, and resistance to host immune responses (40). Studies have shown that NO is related to the pathogenesis of different types of tumors. In tumor patients, elevated NOS expression levels and increased NO content have been detected, and both are highly correlated with the expression of vascular endothelial growth factor (VEGF), angiogenesis, and metastasis (41). NO promotes cell migration by activating focal adhesion kinase (FAK) signaling (42). In pancreatic cancer cells, NO promotes cell invasiveness by activating the phosphatidylinositol 3-kinase (PI3K) and protein kinase B (AKT) signaling pathways, as well as the Ras homolog gene family, member A (RhoA) signaling (43). Genetic instability induced by NO may lead to heterogeneity in tumor cells, which in turn affects tumor development and resistance to treatment (44). The role of NO in cancer is bidirectional. Other studies have found that NO can also inhibit cancer cell proliferation by suppressing these signaling pathways or activating tumor-suppressor pathways (such as the BRCA1/p53 pathway). Low concentrations of NO can promote cancer progression, while high concentrations may lead to cancer cell apoptosis (45).

Arginine can be metabolized by ARG1 and arginine decarboxylase (ADC) to produce polyamines. Polyamines participate in the polyamination modification of proteins, affecting protein activity and promoting cell proliferation (46). Polyamines induce the expression of the transcription factor c-MYC encoded by the myelocytomatosis viral oncogene (c-Myc), thereby inhibiting the expression of p21Cip1 in intestinal epithelial cells (IECs) (47). Removing polyamines in IEC cells induces the expression of the activator protein-1 (AP-1) family member JunD, thereby inhibiting cyclin-dependent kinase 4 (CDK4) transcription (48). Arginine derived from tumor cells stimulates tumor-associated macrophages to biosynthesize polyamines, thereby promoting immune evasion. In addition, polyamines can influence tumor cell migration by affecting the function of Rho GTPases, including RhoA, Rac1, and Cdc42 (49).

Another important metabolic product of arginine, creatine, is an organic acid that helps provide energy to muscles and nerve cells (50). Early studies have shown that creatine and its analog cyclocreatine can inhibit tumor growth and have antitumor potential (51). Recent studies indicate that dietary supplementation with creatine can activate monopolar spindle kinase 1 (MPS1), leading to the phosphorylation of Smad2/3, thereby promoting the expression of downstream target genes, ultimately resulting in the metastasis of colorectal and breast cancer cells (52).

### 2.3 Arginine is a signaling molecule

Arginine can directly bind to arginine-sensing molecules within cells, thereby activating related signaling pathways. The most extensively studied arginine-sensing molecules include the cytosolic arginine sensor for mTORC1 subunit 1 (CASTOR1) of the mammalian target of rapamycin complex 1 (mTORC1), solute carrier family 38 member 9 (SLC38A9), and transfer RNA (tRNA). Research has found that the CASTOR protein is an arginine sensor of the mTORC1 pathway (53). The homodimer CASTOR1 binds arginine and allosterically regulates the adjacent GATOR2 binding site, causing its dissociation from GATOR2, thereby activating downstream mTORC1 (54). Arginine can activate the Rag family of GTPases upstream of mTORC1 through the lysosomal amino acid transporter SLC38A9. SLC38A9 mediates the efflux of various essential amino acids from the lysosome in an arginine-dependent manner, including leucine, which mTORC1 senses through cytoplasmic Sestrin proteins. This protein is crucial for the leucine efflux resulting from protein degradation and the subsequent activation of mTORC1 (55) (**Figure 1**). Arginine regulates the mTOR-mediated synthesis of proteins, lipids, and nucleotides, thereby affecting the proliferation of tumor cells (56). Studies have shown that in pancreatic cancer cells, arginine deprivation inhibits the expression of MMP-1 and MMP-9 and alters the PI3K/Akt pathway, manifested as a reduction in Akt phosphorylation levels (57).

The GCN2 pathway is an important regulatory mechanism for cells to respond to amino acid deficiency. When arginine is lacking, the efficiency of tRNA binding to the ribosome decreases, thereby activating GCN2 signaling, which leads to increased phosphorylation of eukaryotic initiation factor 2α (eIF2α), thus inhibiting protein synthesis within the cell (**Figure 1**). At the same time, activation of GCN2 also upregulates key enzymes in the arginine biosynthesis pathway and the expression of SLC3A2, helping cancer cells maintain arginine supply as much as possible under amino acid-restricted conditions. These changes help cancer cells survive when arginine is deficient (58).

### 2.4 Arginine reprograms metabolism in cancer cells

During the process of normal cells transforming into cancer cells, in order to cope with the demands of rapid cell division and the nutrient-poor microenvironment, tumor cells adjust their metabolic strategies, a process known as metabolic reprogramming. Researchers examined liver cancer cells from mice and patients and found that liver cancer cells accumulate high levels of arginine by increasing arginine uptake and inhibiting its consumption. High concentrations of arginine promote the development of liver tumors. The study also found that arginine binds to RBM39, triggering metabolic reprogramming to promote tumor growth by regulating the expression of metabolism-related genes (1). Under this metabolic reprogramming, tumor cells revert to an undifferentiated embryonic cell state and undergo unlimited division. Therefore, the research team proposed a new therapeutic strategy: targeting cancer-specific arginine-binding factors (such as RBM39) and using Indisulam (a carbonic anhydrase inhibitor) to specifically degrade RBM39, thereby preventing metabolic reprogramming (1). The innovation of this strategy lies in using Indisulam as a molecular glue to ‘stick’ the target protein RBM39 to the cell’s ‘waste disposal system’ (the E3 ubiquitin ligase-proteasome pathway) for targeted degradation. Mechanistically, Indisulam induces a conformational change in the C-terminal domain of RBM39, enabling it to be recognized by the E3 ubiquitin ligase complex DCAF15-DDB1-CUL4. DCAF15 is the key substrate recognition subunit. Indisulam, as a ‘molecular glue, ‘ stabilizes the interaction between RBM39 and DCAF15, leading to the polyubiquitination of RBM39 and its eventual degradation by the proteasome.

## 3 Adequate arginine promotes tumor occurrence and development

**Figure 2:**
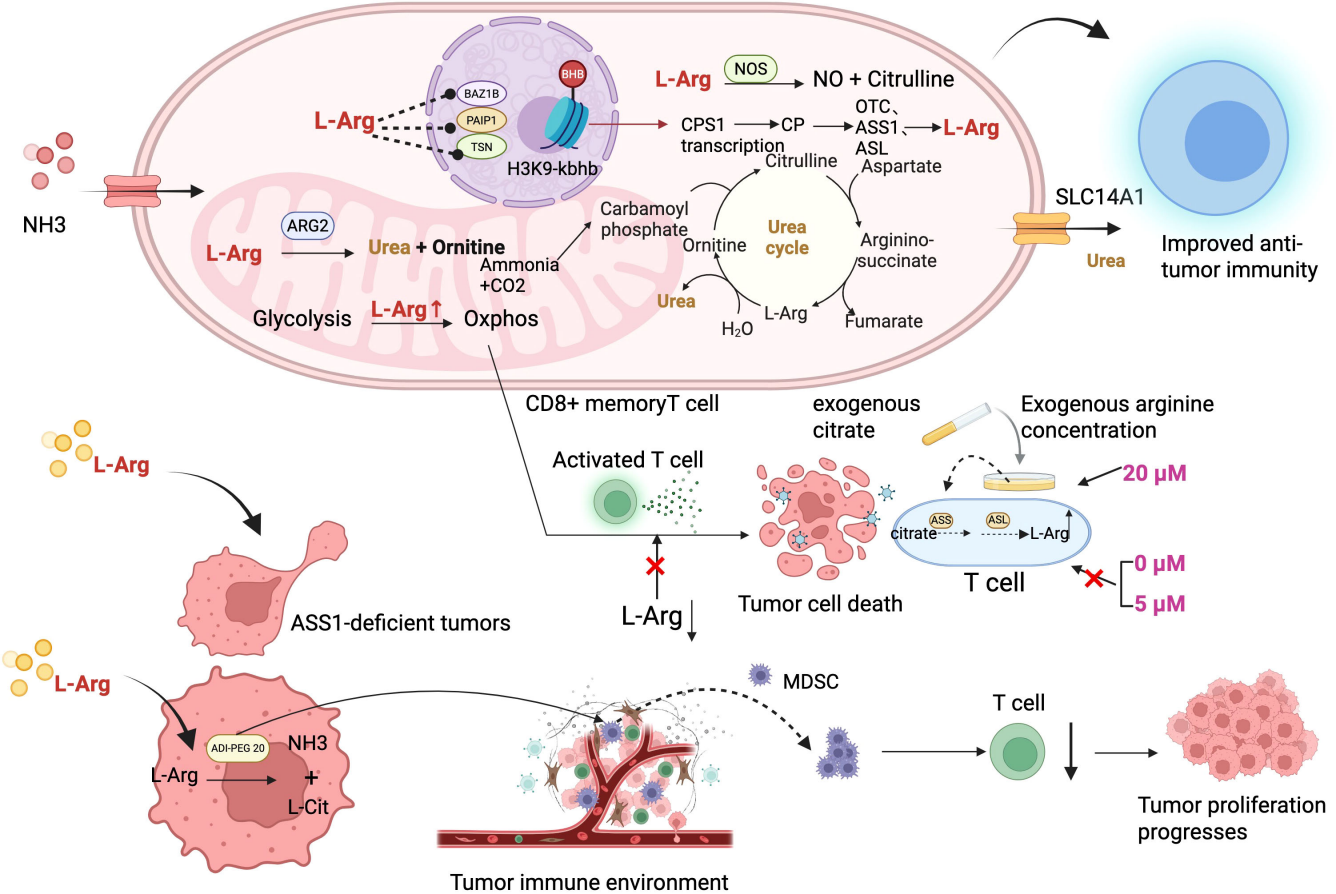
Activation of T cells by arginine and strategies for tumor therapy. The upper part shows the contribution of arginine metabolism in CD8 memory T cells to the survival of these cells and the immune microenvironment. The lower part illustrates inhibiting tumors by activating the tumor microenvironment. Methods to activate the immune microenvironment include supplementing the immune microenvironment with citrate and inhibiting MDSCs (74). When the exogenous arginine concentration is 20 µM, supplementing with citrate can significantly restore T cell proliferation; however, under conditions of 5 µM or 0 µM arginine, citrate cannot restore T cell proliferation (74). L-Arg, L-Arginine; BAZ1B, Bromodomain adjacent to zinc finger domain 1B; PAIP1, Poly(A)-binding protein interacting protein 1; TSN, Tenascin; CPS1, Carbamoylphosphate synthetase 1; OTC, Ornithine transcarbamylase; ASS1, Argininosuccinate synthase 1; ASL, Argininosuccinate lyase; MDSC, Myeloid-derivedsuppressor cell; SLC14A1, Solute carrier family 14 member 1.

Arginine, especially L-arginine, can regulate T cell metabolism, thereby improving T cell survival and their role in anti-tumor immunity. Studies have demonstrated through a series of experiments that active urea and citrulline cycles exist in CD8 memory T cells, which are crucial for enhancing T cell immune memory. It was found that CD8 T cells can synergistically clear ammonia through the urea and citrulline cycles, regulating the CPS1-ARG2 axis to enhance T cell memory and anti-tumor immune capacity (59). Researchers analyzed metabolic and proteomic changes in human primary CD4 T cells activated at different time points using liquid chromatography-mass spectrometry (LC-MS) and found that increasing L-arginine concentration can promote T cells to shift from glycolysis to oxidative phosphorylation (OXPHOS), improve mitochondrial function, enhance oxygen consumption and spare respiratory capacity, provide bioenergetic support for T cells, and thereby enhance their persistence and efficacy in antitumor immunity (60). Further studies have found that the effect of L-arginine on T cell survival is partially mediated through the regulation of nuclear proteins such as BAZ1B, PSIP1, and TSN (61). These proteins exhibit different conformational changes in the presence of L-arginine, and gene editing to delete these genes reduces T cells’ dependency on L-arginine, further supporting the crucial role of L-arginine in T cell survival (61) (**Figure 2**). Overall, L-arginine not only enhances T cell survival by promoting oxidative metabolism, but also improves their anti-tumor response by strengthening the memory phenotype of T cells. This finding provides a new strategy for cancer immunotherapy, particularly in the context of CAR T cell therapy and immune checkpoint inhibition, further exploring the potential of L-arginine as a metabolic regulator.

Arginine is needed for both the activity of immune cells, especially T cells, and the growth of cancer cells. Arginine deprivation therapy has shown anti-tumor potential in the treatment of tumors with ASS1 deficiency or low expression (62). Clinical studies on arginine deprivation have mainly focused on using recombinant arginine deiminase (such as ADI-PEG 20) as a treatment approach. ADI-PEG 20 can convert arginine into citrulline and ammonia, thereby reducing intracellular arginine levels in tumor cells (**Figure 2**). However, there are some challenges in the clinical application of ADI-PEG 20, such as the rapid upregulation of ASS1 in certain tumor cells, which limits the efficacy of arginine deprivation when used alone. In addition, factors such as ASS1 upregulation, autophagy, metabolic cooperation between the tumor stroma and tumor cells, as well as the production of resistance antibodies can affect the effectiveness of arginine deprivation therapy (63). To overcome these problems, researchers have proposed some possible solutions, such as combining arginine deprivation with chemotherapeutic drugs, or using non-immunogenic arginase. Other studies have shown that polyamine metabolism is altered in ASS1-deficient cells. In the absence of arginine, the levels of polyamines in these cells decrease, but polyamine biosynthetic enzymes are compensatorily upregulated. In ASS1-deficient cells, blocking polyamine synthesis with DFMO (an ODC1 inhibitor) significantly increases cell death, providing a therapeutic opportunity for targeting polyamine metabolism (64). The study results also indicate that levels of polyamines (such as putrescine) in plasma can serve as biomarkers to predict patients’ responses to ADI-PEG20. In addition, combining ADI-PEG20 with polyamine synthesis inhibitors (such as DFMO) may provide a synergistic therapeutic approach for ASS1-deficient cancers.

Many cancer cells rely on exogenous arginine for survival, so depleting exogenous arginine has become an effective anti-cancer strategy. Using modified recombinant human arginase [HuArgI (Co)-PEG5000] to remove arginine from the tumor microenvironment enables targeted treatment of pancreatic cancer cells. Research has also revealed that arginine deprivation induces autophagic cell death by activating the autophagy pathway (65). Other research results indicate that pancreatic cancer cells (Panc-1, Capan-1, Hs 766 T, Panc 04.03, and Panc 10.05) exhibit varying degrees of dependence on arginine, and arginine depletion induced by [HuArgI (Co)-PEG5000] can effectively kill these cells (66). There are also studies on the use of recombinant human arginase [HuArgI (Co)-PEG5000] to inhibit the growth of colon cancer cells by depleting arginine. These studies confirmed that arginine deprivation induced by [HuArgI (Co)-PEG5000] can effectively activate autophagy and lead to the death of colon cancer cells (67). This mechanism provides new ideas for the targeted therapy of pancreatic cancer and colon cancer. Under arginine deprivation conditions, in melanoma cells, c-Myc replaces HIF-1α in binding to the E-box in the AS promoter region, promoting the upregulation of AS expression, thereby reducing the therapeutic effect of ADI-based arginine deprivation (68).

Research has found that methylation of the ASS1 promoter is an important predictive marker for the sensitivity of lymphoma cells to arginine deprivation therapy. Treatment combining arginine deprivation with autophagy inhibitors may provide a new therapeutic strategy for lymphoma patients (69). Matthew Fletcher and others focused on studying how L-arginine (L-Arg) depletion suppresses T cell antitumor responses by inducing myeloid-derived suppressor cells (MDSCs), and explored the role of PEGylated arginase (peg-Arg I) therapy in the tumor immune environment (70). The study used mouse models and *in vitro* T cell culture models to investigate the effects of L-arginine depletion on T cell proliferation, metabolism, and survival. The results showed that treatment with peg-Arg I significantly inhibited the proliferation of activated T cells, but did not induce T cell apoptosis or affect the expression of activation markers such as CD25, CD69, and IL-2. It was also found that T cell glycolysis was markedly suppressed, while mitochondrial respiration remained unchanged, indicating that T cells maintain survival through enhanced oxidative metabolism (71). L-arginine deprivation therapy has a dual effect. While this method can effectively inhibit the growth of tumor cells that depend on L-arginine, it can also suppress the anti-tumor response of normal T cells by inducing the accumulation of MDSCs (**Figure 2**). Therefore, when treating cancer patients with peg-Arg I, it is necessary to combine it with strategies that inhibit MDSCs (72). Another study found that α-difluoromethylornithine (DFMO) can reduce the expression and activity of arginase (Arg1) in MDSCs by inhibiting ornithine decarboxylase (ODC) activity, and suppress the CD39/CD73-mediated adenosinergic pathway, thereby restoring T cell-mediated anti-tumor immune responses (73). Werner A and colleagues studied how activated T cells use citrate as a precursor to synthesize arginine, thereby overcoming the inhibition of T cell function caused by arginine deficiency. The researchers explored promoting T cell proliferation in an arginine-deficient environment through exogenous supplementation of citrate, which can be converted into arginine via enzymatic reactions, including the actions of argininosuccinate synthase (ASS) and argininosuccinate lyase (ASL) (74). Studies have found that when the concentration of exogenous arginine is 20 µM, supplementation with citrate can significantly restore T cell proliferation, whereas at arginine concentrations of 5 µM or 0 µM, citrate fails to restore T cell proliferation (74) (**Figure 2**). Research has also found that circulating L-arginine can predict the survival of cancer patients receiving immune checkpoint inhibitors (ICIs) therapy (75). Researchers found through the analysis of multiple ICI clinical trials that the level of L-arginine in patients’ blood can predict their response to ICI immunotherapy (76). This is mainly based on the fact that L-arginine metabolism is an important pathway for regulating immune cells, especially in controlling the activity of T cells. The potential mechanism may be that ICIs activate the immune system, increasing the synthesis and release of L-arginine by immune cells, including T cells, which in turn activates more immune cells, helping the patient survive.

Researchers analyzed the serum metabolite levels of 392 renal cancer patients and found that patients with higher L-arginine levels had significantly longer survival. Clinical trial data showed that patients with high blood ARG levels (ARG ≥42 mM) exhibited better clinical responses, including improved clinical benefit, progression-free survival (PFS), and overall survival (OS) (75). In a PD-1 inhibitor-sensitive MC38 colon cancer mouse model, experimental mice with high baseline ARG levels showed higher tumor rejection rates and longer survival.

## 4 Targeting arginine-metabolizing enzymes and modifications of arginine residues in proteins for cancer therapy

### 4.1 Enzymes of arginine catabolism in tumors

ARGs are key enzymes in arginine metabolism. Increased ARGs activity has been detected in both serum and saliva samples of breast cancer patients, which is significantly associated with the condition and tumor metastasis in cancer patients. They can regulate cancer immune evasion by activating immunosuppressive cells such as MDSCs (77). ARGs are highly expressed in tumor-associated macrophages (TAMs) and myeloid-derived suppressor cells (MDSCs) within the tumor microenvironment (TME), which depletes arginine and leads to impaired T cell activation and proliferation (77). ARG1 can hydrolyze L-arginine to produce urea and ornithine. A study used a mouse liver metastasis model and the CT26 colon cancer cell line to evaluate the effects of ARG1 inhibition on colon cancer cell migration and metastatic colonization, both *in vitro* and in mouse models, by using the ARG1 inhibitor nor-NOHA. It was found that ARG1 enhances the migration ability and metastatic colonization of colon cancer cells by regulating arginine metabolism (78).

**Figure 3:**
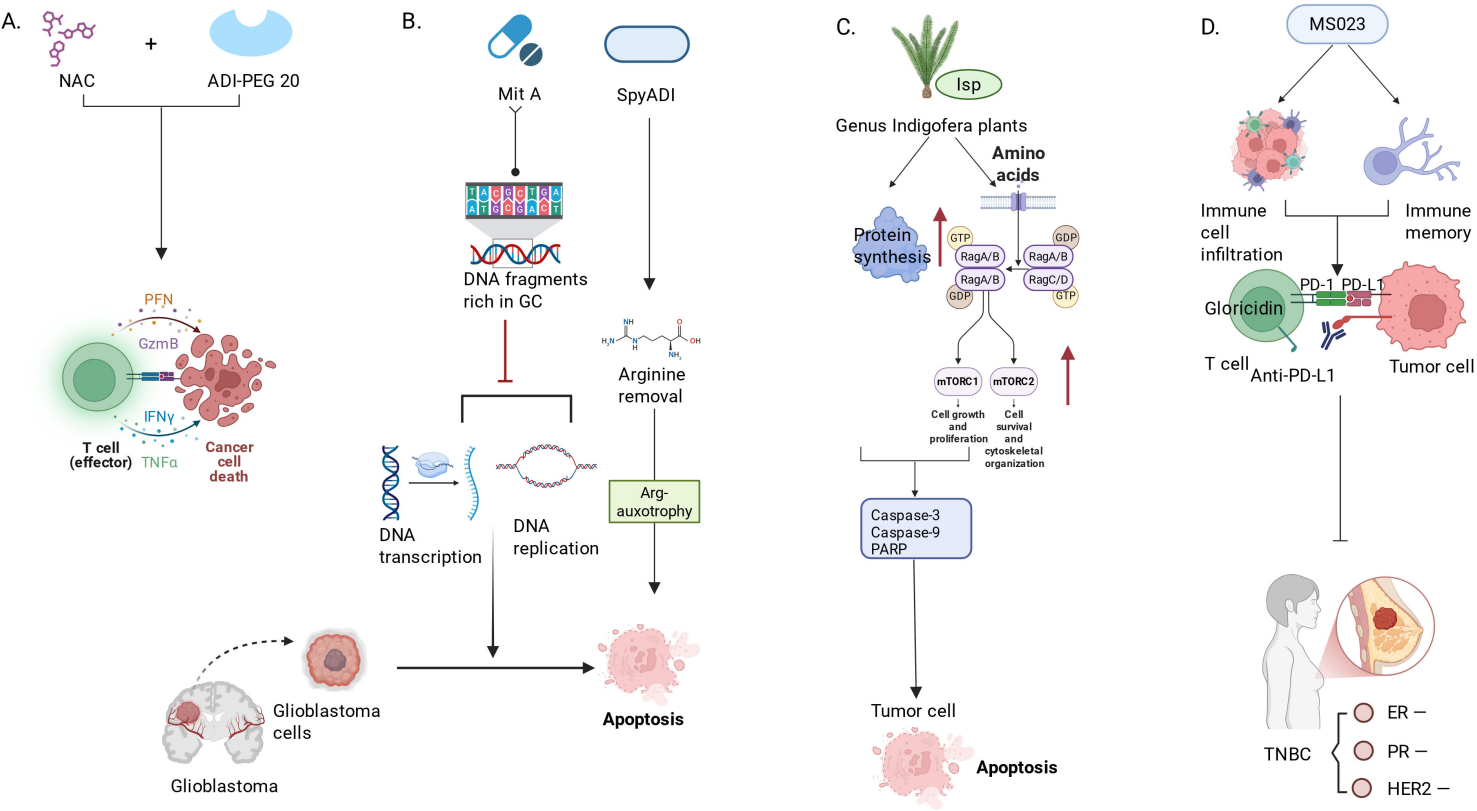
Molecular basis and therapies targeting arginine degradation enzymes and protein arginine modifications in tumors. **(A)** ADI inhibits tumor growth by degrading arginine, but its monotherapy efficacy is limited by tumor adaptability and individual differences. In colon and breast cancer models, NAC was found to enhance the anti-tumor effect of ADI-PEG 20, promote immunogenic cell death, improve mitochondrial function, and activate anti-tumor immune responses. **(B)** SpyADI targets the arginine dystrophic phenotype of GBM by depriving cells of the arginine they need, while MitA exerts antitumor effects by inhibiting Sp1 transcription factors, interfering with DNA transcription, and promoting apoptosis-related pathways. **(C)** Indospicine (Isp), an arginine analogue extracted from the genus Sophora, is combined with arginine deficiency therapy to enhance the therapeutic effect on cancer cells. **(D)** MS023 enhances the response to anti-PD-1 therapy by regulating RNA splicing to reshape the tumor immune microenvironment, promote T cell infiltration, increase TCR diversity, and induce immune memory formation. mTORC1, mammalian Target of Rapamycin Complex 1; mTORC2, mammalian Target of Rapamycin Complex 2; Mit A, Mithramycin A; ATF4, ActivatingTranscription Factor 4.

Arginine deiminase (ADI) is an enzyme that inhibits tumor cell growth by degrading arginine. Researchers have explored enhancing the therapeutic effects of ADI by combining it with other drugs. NAC, as a commonly used antioxidant, has been used in various diseases. Researchers used the MC38 colon cancer mouse model and MDA-MB-231 breast cancer cells to evaluate the effects of ADI-PEG 20 and NAC, alone or in combination, on cell death, cell cycle, mitochondrial function, and characteristics of immunogenic cell death. This study is the first to demonstrate that NAC can enhance the antitumor effects of ADI-PEG 20 and promote immunogenic cell death. This provides a new combination therapy for cancer treatment, particularly for arginine-dependent tumors (79). The combination of ADI-PEG 20 and NAC can enhance the effectiveness of cancer immunotherapy and may be used in conjunction with immune checkpoint inhibitors (such as anti-PD-1/PD-L1) to strengthen anti-tumor immune responses (**Figure 3A**).

Glioblastoma (GBM) is one of the most aggressive brain tumors with a very poor prognosis. Some studies have tested the potential efficacy of combining arginine deiminase from *Streptococcus pyogenes* (SpyADI) with the antitumor antibiotic Mithramycin A (MitA) in treating human glioblastoma (GBM) cells (80). Mithramycin A (MitA) is a highly potent antitumor oligosaccharide antibiotic that mainly inhibits DNA replication and transcription by selectively binding to GC-rich DNA sequences to suppress RNA and DNA polymerases, thereby increasing the expression of cytochrome C, ATF4, and Calnexin, inducing apoptosis, and exerting antitumor activity (81) (**Figure 3B**).

### 4.2 Protein arginine modifications

Studies have found that an environmental pathogen, *Chromobacterium violaceum*, uses its Type III secretion system (T3SS) effector protein CopC to attack caspases by ADPR-deacylation, thereby disrupting host programmed cell death and subsequently suppressing the host immune response. Specifically, CopC can inhibit the enzymatic activity of host cell caspase subunits (such as caspase-7, -8, and -9) by performing ADPR-deacetylation modifications on their arginine residues (82). A *Chromobacterium violaceum* strain lacking CopC showed significantly reduced virulence in a mouse infection model and failed to effectively inhibit the activation of caspase-3, -7 during infection. This study reveals the mechanism by which the bacterial effector protein CopC regulates host cell death. Additionally, CopC and its homologs (such as the OspC protein in Shigella) demonstrate similar functions, further confirming the prevalence and importance of this modification mechanism (82). In addition, bacterial pathogens inject effector proteins into host cells through the type III secretion system (T3SS) to regulate host cell signaling pathways and promote bacterial replication. NleB (an effector protein from enteropathogenic *Escherichia coli*) and SseK1/SseK3 (Salmonella effector proteins) have been shown to modify the death domains (DD) of host TNFR1 and TRADD via arginine-GlcNAcylation, thereby inhibiting death receptor signaling pathways (83). The survival strategies of bacteria offer new targets for cancer therapy.

Indospicine (Isp), an arginine analog derived from plants of the *Indigofera* genus combined with amino acid deprivation therapy, can be used for the treatment of cancer cells. Isp may serve as a potential enhancer aimed at improving the efficacy of arginine deprivation therapy. Regarding the molecular mechanism, Isp further increases endoplasmic reticulum stress and activates apoptosis-related proteins (such as caspase-3, caspase-9, and PARP) by promoting protein synthesis and activating the mTOR pathway, inducing programmed cell death (84) (**Figure 3C**).

Protein arginine methyltransferases (PRMTs) are important protein-modifying enzymes that regulate transcription, splicing, RNA biology, DNA damage responses, and cellular metabolism by adding methyl groups to the arginine residues of target proteins (85). Currently, nine members of the PMRT family have been identified, and numerous studies have found that PMRT5 is closely related to tumor development, especially in prostate cancer, and the regulation of cell death (86). Triple-negative breast cancer (TNBC) is a subtype of breast cancer that lacks estrogen receptors, progesterone receptors, and HER2 receptors, making conventional hormone therapy and HER2-targeted treatments ineffective. Studies have shown that Type-I PRMTs play an important role in antiviral immune responses in triple-negative breast cancer by regulating RNA splicing. Using mouse models, research employing single-cell sequencing and TCR sequencing revealed that the Type-I PRMT inhibitor MS023 enhances the effectiveness of anti-PD-1 immunotherapy by altering the tumor immune microenvironment, promoting T cell infiltration, and facilitating the generation of immune memory. MS023 not only enhances tumor immunogenicity but also effectively increases the proportion of CD8 T cells and promotes TCR diversity, potentially enhancing immune responses through the modulation of RNA splicing (87) (**Figure 3D**). Research results indicate that the combination of MS023 and anti-PD-1 therapy is a promising treatment strategy that can improve the response of TNBC patients to immunotherapy.

## 5 Arg-tRNA loss and modification and protein arginylation in cancer cells

Transfer RNA (tRNA) is a key molecule in decoding the genetic code during translation and is rich in numerous post-transcriptional modifications. Studies have found that arginine-to-cysteine substitutions (R>C substitutants) are significantly enriched in the lung cancer proteome. This enrichment is induced by arginine deprivation, potentially through tRNA misalignments, and is associated with mutations in the KEAP1 pathway. R>C substitutants enhance lung cancer cell resistance to cisplatin (88). Studies have also confirmed that colorectal cancer cells lacking arginine first reduce arginine transfer RNAs, after which ribosomes stall at arginine codons (89). Genes having mutations in arginine codons can help tumor cells overcome arginine deprivation. Therefore, arginine deprivation induces genomic mutations and proteomic changes in cancer cells (89).

**Figure 4:**
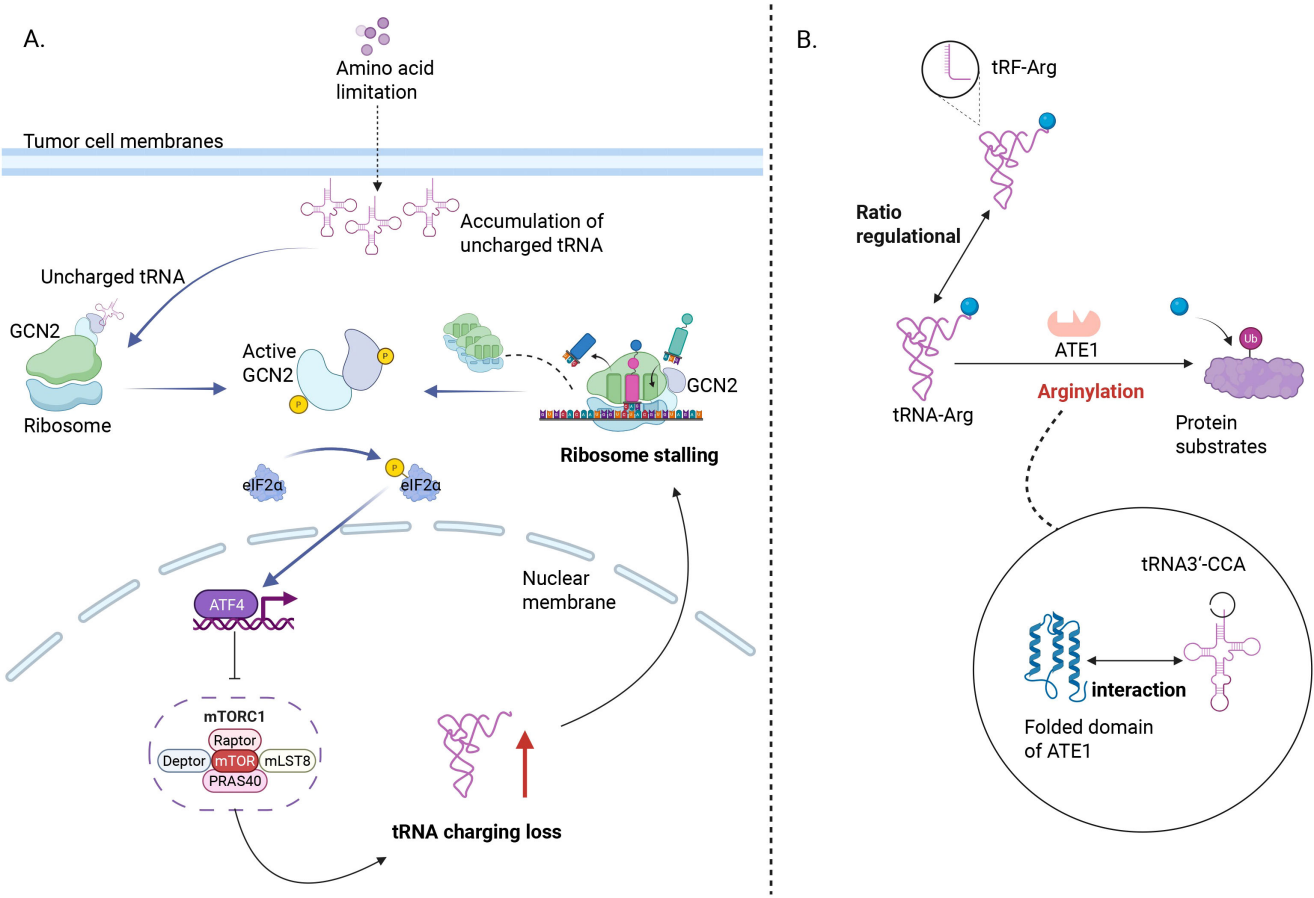
Molecular mechanisms by which cancer cells overcome arginine deprivation and protein arginylation. **(A)** Arginine deprivation in cancer cells causes tRNA misalignment and ribosome stalling, leading to R>C substitutions and proteomic mutations that enhance drug resistance. **(B)** ATE1 catalyzes protein arginylation using tRNA^Arg^, linking translation to protein degradation and cancer regulation. GCN2, General control non-derepressible 2; ATE1, Arginyl-tRNA-protein transferase 1.

Even under conditions of amino acid limitation, translation can be regulated through ribosome restriction. Amino acids can modulate protein synthesis in mammalian cells through a mechanism known as ‘ribosome pausing.’ Darnell and colleagues primarily studied the responses of the mTORC1 and GCN2 pathways to tRNA charging and ribosome stalling under conditions of arginine and leucine deficiency. Their findings showed that mTORC1 and GCN2 can relieve the loss of tRNA charging and ribosome stalling caused by leucine deprivation; however, these pathways were ineffective against the loss of tRNA charging and ribosome stalling induced by arginine deprivation (81) (**Figure 4**).

Arginyl-tRNA-protein transferase 1 (ATE1) primarily functions to transfer arginine to the N-terminal aspartate or glutamate residues of proteins, facilitating their degradation via the ubiquitin pathway. Abeywansha and colleagues conducted an in-depth study of the structure of ATE1 from *Saccharomyces cerevisiae* and its interaction with tRNA^Arg^. ATE1 can perform the arginylation reaction through contacts between its GNAT fold domain and the groove of the tRNA acceptor stem, as well as the 3’-CCA end (90). Protein arginylation is catalyzed by the enzyme ATE1, which uses tRNA^Arg^ as the amino acid donor. Since the use of tRNA^Arg^ competes with protein synthesis, the specificity of ATE1 during translation becomes an important issue (**Figure 4**). Lan focused on the three-dimensional structure of human ATE1 in complex with its substrate (91). This provides a new perspective for understanding the complex regulatory mechanisms of protein arginylation, especially the interplay between translation and modification, and also reveals its potential as a therapeutic target in cancer.

METTL1 is an RNA methyltransferase of the METTL family that catalyzes m7G modification in tRNA and is involved in cell cycle regulation and the translation of cancer-related genes. Studies have found that METTL1 is overexpressed in various cancers and is associated with poor prognosis. It was revealed that METTL1 enhances mRNA translation efficiency by catalyzing m7G modification of tRNA (especially Arg-TCT-4-1), particularly increasing the translation efficiency of mRNAs for cell cycle regulators that are rich in AGA codons. Arg-TCT is upregulated in many cancer cells and promotes oncogenic transformation, providing a new target for precise cancer therapy (92).

## 6 Targeting the arginine metabolic pathway in cancer patients

One of the mechanisms of immune evasion in cancer is through immune checkpoint inhibition, particularly the interaction between PD-1 and PD-L1. PD-L1 is overexpressed on the surface of many types of cancer cells and tumor-infiltrating immune cells, helping tumor cells evade T cell immune surveillance and limiting the function of the immune system (93). In recent years, the clinical application of immune checkpoint inhibitors (such as pembrolizumab) has significantly changed the landscape of cancer treatment. However, the survival of tumor cells also involves other mechanisms, among which autophagy, as a cellular self-protection mechanism, plays a dual role in the growth and survival of tumor cells. Robainas M. and colleagues focused on exploring the interaction between the PD-1/PD-L1 pathway and autophagy in tumor immune evasion and therapy, finding that PD-L1 helps tumor cells survive under immune pressure by activating autophagy, especially in nutrient-deprived and hypoxic tumor microenvironments. The study also found that in multiple mouse tumor models, the combination of immune checkpoint inhibitors and autophagy inhibitors exhibited significant antitumor activity (94).

Arginine deficiency in the tumor microenvironment can weaken the expression of CTLA4 and PD-1 on activated T cells, thereby inhibiting the proliferation of activated T cells (95). Supplementing arginine *in vitro* is also considered a potentially valuable anti-tumor therapeutic strategy, but arginine supplementation carries the risk of promoting tumor cell proliferation. Specifically increasing the concentration of arginine within T cells may potentially address this issue. For example, stably expressing ASS1 and OTC in T cells and cooperating with different chimeric antigen receptors (CARs) can enable the recycling of ornithine into arginine, thereby enhancing the antitumor function and survival of CAR-T cells (96).

Given the complexity of arginine requirements among different immune cells in tumor cells and the tumor microenvironment, specifically targeting the arginine transport process on cells is a feasible strategy (97). The arginine transporter SLC7A1 is involved in the progression of hepatoblastoma and ovarian cancer (98). The expression of the SLC7A2 gene has been identified as an independent prognostic biomarker for breast cancer (99), and its low expression enhances multidrug resistance in non-small cell lung cancer (NSCLC) (100), reduces immune infiltration, and leads to poor prognosis (101). Related studies have indicated that SLC7A3 is a prognostic biomarker for breast cancer (102). These pieces of evidence collectively suggest that targeting arginine transport to block tumor cells from utilizing exogenous arginine is a potentially effective antitumor strategy.

Research by Cook and colleagues demonstrated that immunization with citrullinated ENO1 peptide segments can induce a strong Th1 response. Citrullinated ENO1 peptide segments can be used to treat melanoma, pancreatic cancer, and lung cancer. The study results indicate that citrullinated antigens can effectively activate the immune system and enhance anti-tumor immune responses, particularly when tumor cells undergo stress responses such as nutrient deprivation. The study also shows that citrullinated ENO1 is effective not only under specific HLA types but also in individuals with different HLA types, making it a candidate vaccine with broad clinical application potential (103).

**Figure 5:**
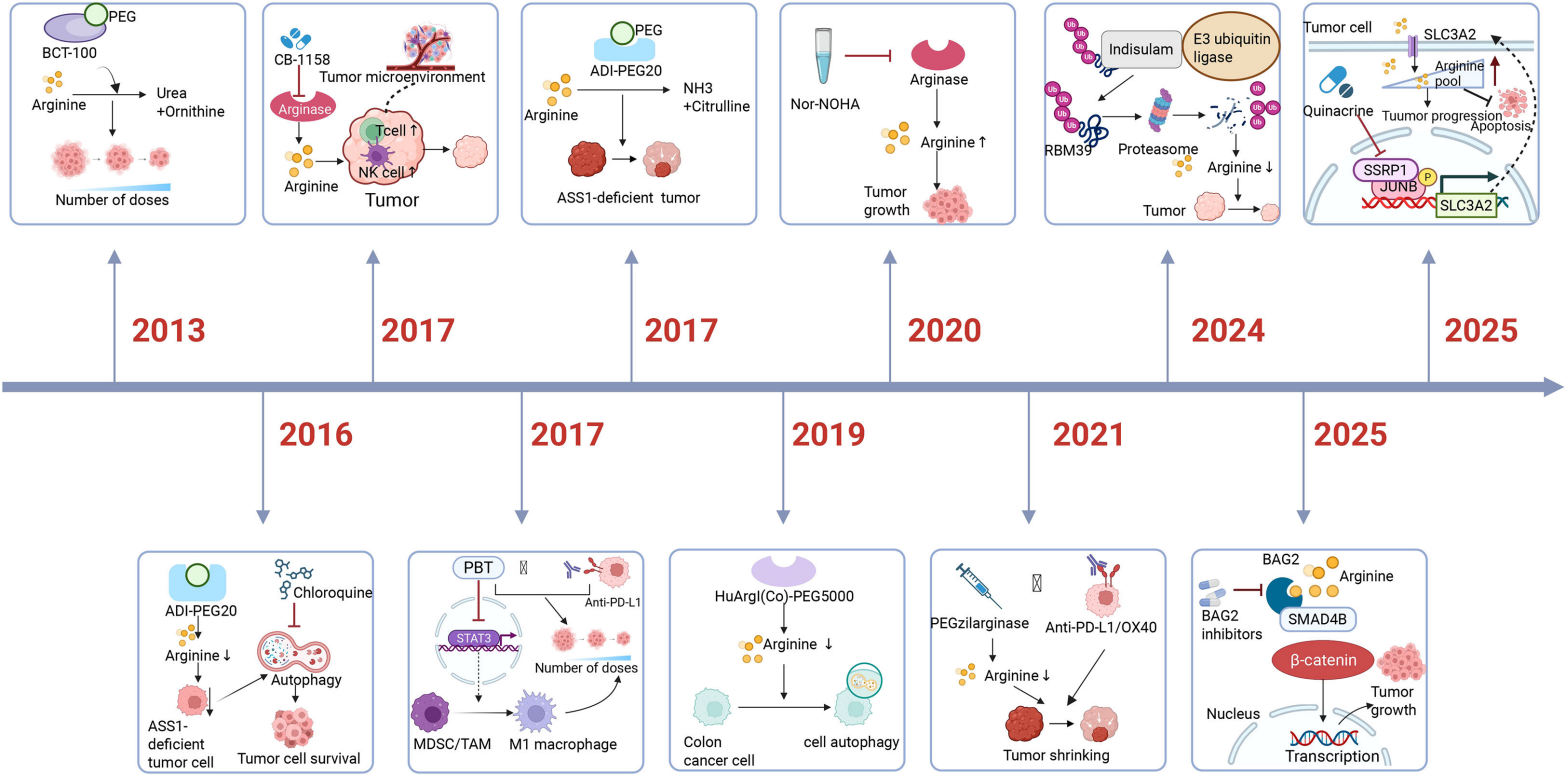
The history of tumor therapy targeting the arginine metabolism pathway.

ADI-PEG 20 (PEGylated arginine deiminase) is an enzyme that depletes arginine by converting it into citrulline and ammonia (104) (**Figure 5**). It is currently used for malignant pleural mesothelioma, hepatocellular carcinoma (for which Phase III clinical trials have been conducted), melanoma, non-small cell lung cancer (NSCLC), acute myeloid leukemia (AML), and uveal melanoma (105). This enzyme achieved longer PFS (106) and other clinical outcomes in Phase I and II clinical trials (107). Brin E. focused on studying the role of ADI-PEG 20 in the tumor immune microenvironment and found that ADI-PEG 20 not only regulates immune checkpoints by affecting PD-L1 expression on tumor cells, but also improves the tumor immune microenvironment by reducing Treg cell accumulation and promoting T cell infiltration (108). This provides a new perspective for future cancer immunotherapy, especially in tumor types that exhibit immune evasion, where ADI-PEG 20 may become an effective immunomodulator.

In addition, a modified human arginase-1 (pegzilarginase) that can deplete systemic arginine shows significantly better tumor growth control when combined with immunotherapy drugs such as anti-PD-L1 or agonist anti-OX40 than with monotherapy (109). It also enhances the activity of CD8 T cells, aiding immune-mediated tumor clearance. This new finding provides a promising strategy for treating cancers that depend on exogenous arginine and for improving the efficacy of immunotherapy. The combination of pegzilarginase and immunotherapy enhances antitumor immune responses by increasing CD8 T cells and promoting the polarization of tumor-associated macrophages toward the M1 type. BCT-100 (Pegylated Recombinant Human Arginase 1) is a recombinant human arginase 1 that, after PEGylation, reduces immunogenicity and prolongs its half-life. It depletes arginine by hydrolyzing it into ornithine and urea, thereby exerting an antitumor effect (**Figure 5**). Currently mainly used to explore treatments for hepatocellular carcinoma (HCC) (110) and melanoma (111). *In vitro* and mouse models showed that BCT-100 can significantly reduce arginine levels in bladder cancer, inhibit the AKT/mTOR pathway, induce autophagy and apoptosis, and suppress tumor growth (112).

Research by Cao Y. and colleagues using a 4T1 breast cancer mouse model showed that both L-arginine and docetaxel (DTX) alone could significantly inhibit tumor growth, while the combination of L-arginine and docetaxel exhibited the strongest tumor-suppressing effect. The combined treatment of DTX and L-Arg was able to significantly enhance the anti-tumor immune response in mice, increase immune cell infiltration in the tumor microenvironment—particularly dendritic cells and effector T cells—and reduce the proliferation of MDSCs (113). This study provides a new potential strategy for the treatment of breast cancer. By combining L-arginine and docetaxel, it not only directly inhibits tumor growth but also activates anti-tumor immune responses by modulating the immune system.

The arginase inhibitor CB-1158 (INCB001158) selectively inhibits arginase activity (mainly Arg1) in the tumor microenvironment, increases arginine levels in the TME, and enhances the activity of tumor-infiltrating CD8 T cells and NK cells, thereby improving anti-tumor effects (114) (**Figure 5**). It is currently applicable to various solid tumors, including colorectal cancer (MSS CRC), NSCLC, and melanoma, and is often used in combination with PD-1/PD-L1 inhibitors. The combination therapy of Retifanlimab and CB-1158 has a relatively high safety profile (115). Nor-NOHA is a small molecule arginase inhibitor, primarily studied *in vitro* or in animal models to investigate the effects of ARG inhibition on tumor or immune cells (116). To cope with the effects caused by ADI-PEG20, tumor cells initiate the autophagy process to replenish arginine deficiency, thereby allowing tumor cells to survive. Therefore, the combined use of ADI-PEG20 and the autophagy inhibitor chloroquine can effectively suppress tumor growth (117).

Peripheral T-cell lymphoma (PTCL) is a highly heterogeneous malignancy that is often associated with poor prognosis. PTCL cells rely on the arginine transporter SLC3A2 for the uptake of exogenous arginine, which in turn supports tumor cell proliferation and immune evasion. Research found that SSRP1 acts as a transcriptional co-activator and, together with JUNB, regulates the transcription of SLC3A2. Quinacrine inhibits the transcription and expression of SLC3A2 by targeting SSRP1, thereby reducing intracellular arginine levels, suppressing PTCL cell proliferation, and inducing apoptosis. Therefore, quinacrine and histone deacetylase inhibitors are considered effective therapeutic candidate drugs (118).

The GCN2-eIF2α-ATF4 pathway has been identified as the classical pathway responding to amino acid deficiency (119). Recently, through high-throughput screening, Chen et al. discovered that BAG2 acts as an arginine-sensing protein, and its glutamine residue 167 binds to arginine. The study revealed that when arginine is abundant, BAG2 binds to SAMD4B, inhibiting β-catenin degradation and promoting cell proliferation; when arginine is deficient, BAG2 releases SAMD4B, which then binds to β-catenin and promotes its degradation, thereby enhancing ATF4 stability and supporting cell survival. Further research found that the BAG2-SAMD4B pathway works in concert with the classical GCN2-eIF2α-ATF4 pathway to collectively regulate the stress response, helping tumor cells cope with the stress caused by arginine deprivation (120). This study provides a new approach, suggesting that treatment with arginine deprivation combined with an ATF4 inhibitor (such as ATF4-IN-1) may effectively suppress tumor cell growth.

The tumor microenvironment (TME) is a tissue composed of tumor cells, immune cells, and other cells and molecules, characterized primarily by immunosuppression and metabolic reprogramming. Scientists from the Chinese Academy of Sciences have revealed a novel mechanism in which breast cancer cells lead to pro-tumor polarization of tumor-associated macrophages (TAMs) through providing arginine, thereby helping tumor cells evade immune attacks (5). Mechanistically, polyamines produced from arginine metabolism enhance pro-tumor TAM polarization through DNA demethylation mediated by thymine DNA glycosylase (TDG), while the interaction between breast cancer cells and TAMs diminishes the ability of arginine to increase CD8 T cell activity. Targeting the arginine-polyamine-TDG axis can effectively inhibit breast cancer cell growth.

In 2013, it was discovered that BCT-100, after PEGylation, can hydrolyze arginine into ornithine and urea; In 2016, it was found that in ASS1-deficient tumors, arginine depletion induces nutrient starvation through ADI-PEG20, chloroquine inhibits autophagy, and the combination of the two can enhance cell death; In 2017, CB-1158 was found to selectively inhibit arginase activity (mainly Arg1) in the tumor microenvironment, and thereby improving anti-tumor effects; In 2017, a new drug PBT (composed of DFMO and Trimer PTI) was found to inhibit polyamine metabolism, significantly suppressing tumor growth;In 2017, it was discovered that ADI-PEG 20, after PEG modification, can deplete arginine in the tumor microenvironment; In 2019, Human recombinant arginase I (Co)-PEG5000 [HuArgI (Co)-PEG5000] could effectively activate autophagy and lead to colon cancer cell death; In 2020, Nor-NOHA was identified as a small molecule arginase inhibitor; In 2021, pegzilarginase was used in combination with immunotherapy drugs such as anti-PD-L1 or anti-OX40; In 2024, Indisulam has been found to inhibit liver cancer cells by degrading RBM39 through the E3 ubiquitin ligase-proteasome pathway; In 2025, it was discovered that intervening in the BAG2-SAMD4B pathway could potentially enhance the effects of tumor arginine deprivation therapy; In 2025, it was discovered that quinacrine inhibits the transcription and expression of SLC3A2 by targeting SSRP1, thereby reducing intracellular arginine levels, inhibiting PTCL cell proliferation and inducing apoptosis.

## 7 Discussion and conclusion

Arginine is a nutrient required for the development and survival of various tumors. Naturally, depleting arginine within tumor cells has become one of the methods for treating tumors. In oncology, this method is referred to as arginine deprivation therapy (121). However, arginine deprivation therapy faces inherent bottlenecks, such as weakening the function of immune cells in the body. We discussed in detail how to use arginine deprivation therapy while enhancing the body’s immune function, especially the activity of T cells.

With the elucidation of the molecular mechanisms of arginine metabolism and the molecular mechanisms that maintain high intracellular levels of arginine in cancer cells, our review not only summarizes the application and molecular mechanisms of arginine deprivation therapy in tumors (122), but also reviews and discusses the synthesis and modification of tRNA-Arg, which are gradually becoming hot targets in cancer treatment. At the same time, effector proteins from microorganisms and metabolites from plants (123), together with arginine deprivation therapy, have become effective means of killing tumor cells (124). The molecular mechanisms and applications of arginine deprivation therapy and other therapies, such as immunotherapy, are also well discussed and analyzed in this article.

Our research has more thoroughly advanced arginine deprivation therapy from both molecular mechanisms and applications. Our review can better provide methods and guidance for clinicians in treating cancers caused by arginine metabolism disorders.
